# Bioavailability and dose-dependent anti-tumour effects of 9-*cis* retinoic acid on human neuroblastoma xenografts in rat

**DOI:** 10.1054/bjoc.2001.2186

**Published:** 2001-12

**Authors:** F Ponthan, P Kogner, P Bjellerup, L Klevenvall, M Hassan

**Affiliations:** Childhood Cancer Research Unit, Dept of Women's and Children's Health, Karolinska Institutet, Karolinska Hospital, Stockholm, S-171 76, Sweden; Dept of Clinical Chemistry, Dept of Haematology, Huddinge University Hospital, Stockholm, S-141 86, Sweden

**Keywords:** vitamin A, retinoic acid, neuroblastoma, apoptosis, differentiation, nude rat

## Abstract

Neuroblastoma, the most common extracranial solid tumour in children, may undergo spontaneous differentiation or regression, but the majority of metastatic neuroblastomas have poor prognosis despite intensive treatment. Retinoic acid regulates growth and differentiation of neuroblastoma cells in vitro, and has shown activity against human neuroblastomas in vivo. The retinoid 9-*cis* RA has been reported to induce apoptosis in vitro, and to inhibit the growth of human neuroblastoma xenografts in vivo. However, at given dosage, the treatment with 9-*cis* RA caused significant toxic side effects. In the present study we investigated the bioavailability of 9-*cis* RA in rat. In addition, we compared two different dose schedules using 9-*cis* RA. We found that a lower dose of 9-*cis* RA (2 mg day^−1^) was non-toxic, but showed no significant effect on tumour growth. The bioavailability of 9-*cis* RA in rat was 11% and the elimination half-life (t1/2) was 35 min. Considering the short t1/2, we divided the toxic, but tumour growth effective dose 5 mg day^minus;1^ into 2.5 mg p.o. twice daily. This treatment regimen showed no toxicity but only limited effect on tumour growth. Our results suggest that 9-*cis* RA may only have limited clinical significance for treatment of children with poor prognosis neuroblastoma. © 2001 Cancer Research Campaign http://www.bjcancer.com


*British Journal of Cancer* (2001) **85**(12), 2004–2009
© 2001 Cancer Research Campaign
doi: 10.1054/ bjoc.2001.2186, available online at http://www.idealibrary.com on IDEAL

http://www.bjcancer.com

### Bioavailability and dose-dependent anti-tumour effects of 9-*cis* retinoic acid on human neuroblastoma xenografts in rat

**F Ponthan[1], P Kogner[1], P Bjellerup[2], L Klevenvall[1] and M Hassan[3]**

[1]Childhood Cancer Research Unit, Dept of Women's and Children's Health, Karolinska Institutet, Karolinska Hospital, S-171 76 Stockholm, Sweden; [2]Dept of Clinical Chemistry; [3]Dept of Haematology, Huddinge University Hospital, S-141 86 Stockholm, Sweden

**Summary** Neuroblastoma, the most common extracranial solid tumour in children, may undergo spontaneous differentiation or regression, but the majority of metastatic neuroblastomas have poor prognosis despite intensive treatment. Retinoic acid regulates growth and differentiation of neuroblastoma cells in vitro, and has shown activity against human neuroblastomas in vivo. The retinoid 9-*cis* RA has been reported to induce apoptosis in vitro, and to inhibit the growth of human neuroblastoma xenografts in vivo. However, at given dosage, the treatment with 9-*cis* RA caused significant toxic side effects. In the present study we investigated the bioavailability of 9-*cis* RA in rat. In addition, we compared two different dose schedules using 9-*cis* RA. We found that a lower dose of 9-*cis* RA (2 mg day$^{-1}$) was non-toxic, but showed no significant effect on tumour growth. The bioavailability of 9-*cis* RA in rat was 11% and the elimination half-life ($t_{1/2}$) was 35 min. Considering the short $t_{1/2}$, we divided the toxic, but tumour growth effective dose 5 mg day$^{-1}$ into 2.5 mg p.o. twice daily. This treatment regimen showed no toxicity but only limited effect on tumour growth. Our results suggest that 9-*cis* RA may only have limited clinical significance for treatment of children with poor prognosis neuroblastoma. © 2001 Cancer Research Campaign http://www.bjcancer.com

**Keywords**: vitamin A; retinoic acid; neuroblastoma; apoptosis; differentiation; nude rat

Vitamin A and its analogue, retinoic acid (RA), play an important role in normal cellular differentiation and programmed cell death (Sucov and Evans, 1995). Retinoids signal through two sets of closely related intracellular receptors, RAR (retinoic acid receptor α, β, γ) and RXR (retinoic X receptor α, β, γ), which all belong to the steroid receptor super family (Sucov and Evans, 1995). All-*trans* RA (ATRA) binds to RAR with high affinity but does not bind to RXR, whereas 9-*cis* RA, an isomer of ATRA, binds and transactivates both RARs and RXRs (Heyman et al, 1992; Redfern et al, 1995). 9-*cis* RA is mainly metabolized in the liver by cytochrome P450 (CYP 450) to its oxidized form 4-oxo-9-*cis* RA (Shirley et al, 1996; Howell et al, 1998).

Neuroblastoma is the most common extracranial solid tumour in children. It is characterized by a diversity of clinical behaviour, ranging from spontaneous remission to rapid tumour progression and death. Most metastatic neuroblastomas show progression and poor clinical outcome despite intensive multimodal therapy. This increases the importance of finding new therapeutic drugs.

Retinoic acid has been reported to induce differentiation of neuroblastoma cells in vitro (Sidell, 1982; Reynolds et al, 1991; Abemayor, 1992; Redfern et al, 1995; Lovat et al, 1997). Recently, a clinical study from the Children's Cancer Group (CCG) showed significantly improved event-free survival for children with high-risk neuroblastoma when treated with an intermittent schedule of high-dose 13-*cis* RA after autologous bone marrow transplantation (Matthay et al, 1999). 9-*cis* RA has been shown to induce apoptosis in vitro in neuroblastoma cells, using a short-term dosing schedule of 5 days treatment and subsequent washout and reincubating in RA-free medium (Lovat et al, 1997). Animal studies on breast cancer have showed promising results using 9-*cis* RA either as a single agent or in combination with tamoxifen (Anzano et al, 1994).

In a previous study, we treated nude rats with human neuroblastoma xenograft tumours with 9-*cis* RA 5 mg day$^{-1}$ (Ponthan et al, 2001). This experiment showed that 9-*cis* RA could induce a significant reduction in tumour growth, but with severe toxic side effects. The present study was designed to investigate the bioavailability of 9-*cis* RA in rat. In addition, we wanted to compare two different dose schedules using 9-*cis* RA, in order to find a non-toxic but still tumour-effective treatment regimen.

### MATERIALS AND METHODS

### Chemicals

The retinoids used in this study 9-*cis* retinoic acid (Ro 04-4079), 4-oxo-9-*cis* retinoic acid (Ro 47-8078) and 13-*cis* acitretin (Ro 13-7652) were kindly provided by Hoffmann-La Roche (Basel, Switzerland). The compounds were dissolved in methanol and the stock solutions were stored at –70˚C. The internal standard (IS), 13-*cis* acitretin 3 µg ml$^{-1}$, was prepared in aliquots and stored at –70˚C. Working solutions of the retinoids for HPLC were obtained by sequential dilutions of the respective stock solutions in methanol at the time for analysis. Methanol, acetonitrile and tetrahydrofuran were of HPLC grade and were supplied, together with all other analytical grade reagents (glacial acetic acid, diethyl ether, ethyl acetate) and buffers, from Merck (Darmstadt Germany). Water for HPLC was prepared by a Milli-Q-Water

*Received 21 June 2001*
*Revised 17 September 2001*
*Accepted 20 September 2001*

*Correspondence to:* F Ponthan purification system. All handling of retinoids and biological samples was performed protected from light. Plasma samples were stored at –70˚C for less than 3 weeks before analysis.

### Pharmacokinetic study

Male Sprague-Dawley rats (B&K Universal, Sollentuna, Sweden) with the average weight of 250 g were given a single intravenous or oral dose of 9-*cis* RA 30–45 mg $kg^{-1}$. 9-cis RA was dissolved in DMSO (Sigma, St Louis, USA) to the concentration of 60 mg $ml^{-1}$, and then diluted 1:10 (v/v to 6 mg $ml^{-1}$) in phosphate-buffered saline (Gibco Brl, Paisley, Scotland, UK) with 1% of fetal bovine serum (Gibco Brl), before 1.5 ml was orally or intravenously administrated.

### Blood collection and extraction procedures

Blood was collected by cardiac exsanguination in tubes containing sodium heparin (100 IU $ml^{-1}$, 0.05 ml $ml^{-1}$ whole blood, Karolinska Pharmacy, Sweden). Two animals were sacrificed at each time point. Samples were collected from untreated animals and from post injection of 9-*cis* RA (15 min to 8 hours). The blood was immediately centrifuged and plasma was stored at –70˚C until analysis, which was performed within 3 weeks from sampling.

In a 10 ml glass tube, 25 µl of the internal standard was added. After addition of 0.5 ml plasma and 0.1 ml phosphate buffer, pH 7, (0.025 M $KH_2PO_4$ and 0.04 M $Na_2HPO_4*2H_2O$) the compounds were extracted for 5 min with 3 ml of a diethyl ether-ethyl acetate (50/50, v/v) mixture by vortex mixing. After centrifugation at 2000 *g* for 10 min at 4˚C, the organic phase was evaporated to dryness. The residue was dissolved in 90 µl methanol and transferred to an injection vial with cap, for HPLC analysis.

### Chromatographic conditions

HPLC analysis was performed using a LKB 2150 pump equipped with an auto sampler (Perkin-Elmer ISS-100) and a variable-wavelength UV detector Spectromonitor (LDC/Milton Roy). The analytical column, a Prodigy ODS (3) silica column (150 × 4.6 mm) with 3 µm particles (Phenomenex, California, USA) was fitted with a guard column (Nova-Pak C18, Waters, USA). Data were acquired and analysed using System Gold (Beckman Instruments Inc). An isocratic gradient was prepared with the final composition of 52.87% methanol, 28.47% acetonitrile, 16.66% water, 1.66% tetrahydrofuran and 0.34% acetic acid. The mobile phase was degassed by ultrasonic treatment before HPLC analysis. The flow rate was 1 ml $min^{-1}$ and the UV detection was carried out at 350 nm. A 65 µl aliquot of each sample was auto-injected and data were collected during 30 min. The total time between injections was 32 min.

### Calibration and system validation

Standard curves were prepared in plasma, covering the expected retinoid concentrations. Control plasma was spiked with known concentrations of 9-*cis* RA, 4-oxo-9-*cis* RA and internal standard (13-*cis* acitretin) in the range of 0.01 to 25.6 µg $ml^{-1}$ plasma, in duplicate. The recovery was determined in duplicate and calculated from peak heights, of non-extracted and extracted samples of 9-*cis* RA and 4-oxo-9-*cis* RA. Calibration graphs and analysis of linearity were performed by linear least-squares regression analysis, plotting peak-height ratios of the compound and the internal standard against the concentration of the compound. We used an external standard containing known concentrations of the retinoids and IS to check the day-to-day and the within-day reproducibility and variation, concerning the retention times and the peak heights.

### Neuroblastoma cells

The adrenergic neuroblastoma cell line SH-SY5Y was kindly provided by Dr June Biedler, The Memorial Sloan-Kettering Cancer Center, New York (Biedler et al, 1973). The cells were grown at 37˚C in a humidified 95% air/5% $CO_2$ atmosphere. Eagle’s minimum essential medium was supplemented with 10% fetal bovine serum, L-glutamine 2 mM, penicillin G 100 IU $ml^{-1}$ and streptomycin 100 µg $ml^{-1}$ obtained from Gibco Brl. The medium was changed twice a week and confluent cultures were subcultivated after treatment with 0.5 g $l^{-1}$ trypsin and 0.2 g $l^{-1}$ EDTA (Gibco Brl). Cultures were free from mycoplasma as detected by DNA staining. For subcutaneous injections, a single cell suspension, $100 \times 10^6$ cells $ml^{-1}$, was prepared in culture medium supplemented with L-glutamine (2 mM). The viability and the cell concentration were calculated by trypan blue dye exclusion using a haematocytometer.

### Nude rats

21 male nude rats (HsdHan: RNU-rnu, Harlan Netherlands) were used for xenografting at the age of 5–6 weeks with an approximate weight of 150–200 g. No animal was excluded from the study, with the exclusion criteria applied, namely development of an open wound over the tumour.

### Ethics

The experiments described herein were approved by the regional ethics committee for animal research. They also met the ethical standards required by the United Kingdom Co-ordinating Committee on Cancer Research (UKCCCR) Guidelines (1998).

### Xenografting

Establishment of neuroblastoma xenografts was performed as previously described (Nilsson et al, 1993). In short, animals were anaesthetised with Hypnorm (Janssen Pharmaceutica, Beerse, Belgium). Twenty million cells suspended in 0.2 ml medium were injected in each hind leg. At injection a 23-gauge cannula was used to deposit the suspension subcutaneously. The procedure was carefully performed, not to pierce the muscle fascia, and not to lose cells by leakage from the injection site. A small delineated wheal appeared at the injection site.

### Quantification of tumour growth

When tumour take was evident on palpation and/or visible, the tumour length (along the tumour long axis) and width (perpendicular to the long axis) were measured with a calliper every second day. Tumour volume was calculated by length × $width^2$ × 0.44 (Wassberg et al, 1997). The true tumour weight was recorded at autopsy. Tumour volume index was calculated using the measured volume divided with the volume measured at tumour take at start of treatment.

*© 2001 Cancer Research Campaign*

### Retinoid treatments

When a tumour in an animal reached a volume of 0.3 ml (designated day 0), the rat was randomised into one of two groups, and treatment was started. Only those tumours that had reached a volume of 0.3 ml when treatment was started were followed and evaluated for response. All treatments were administered with a gastric feeding tube. In the first set of rats, 7 animals (10 tumours) were continuously treated orally during 12 days with 2 mg of 9-*cis* retinoic acid suspended in 1.5 ml of peanut oil. Another 7 animals (11 tumours) received 1.5 ml peanut oil for 12 days, as a control vehicle.

In the following therapeutic experiment a second set of rats, 4 animals (3 tumours, one rat did not develop tumours within reasonable time) were continuously treated orally twice a day during 10 days with 2.5 mg of 9-*cis* retinoic acid suspended in 1.5 ml of peanut oil. The time gap between the administrations was 8–12 hours. Another 4 animals (4 tumours), received 2 × 1.5 ml peanut oil for 10 days, as a control vehicle. Hence, in order to have a fully comparable control group for each treatment group, there were 2 control groups necessary. All animals were monitored for signs of toxicity including weight loss, yellowness and diarrhoea during treatment.

### Statistics and pharmacokinetic analysis

Statistical analysis was performed using Mann–Whitney U test for 2 independent samples and Kruskal–Wallis test with multiple comparisons for more than 2 groups. Concentration–time data for 9-*cis* RA and its metabolite (4-oxo-9-*cis* RA) were adjusted to a one-compartment open model using Gauss–Newton (Levenberg–Hartly) criteria. Parameters such as the distribution volume of the central compartment, the elimination rate constant, the plasma maximum concentration and the microconstants were estimated. Whereas, the clearance (CI) and the distribution volume at steady-state were calculated from the primary parameters. The area under the plasma concentration versus time curve (AUC) was calculated from the model-derived parameters and the elimination half-life was calculated from the slope of the phase of elimination. The pharmacokinetic modelling was performed using WinNonlin version 3.0 (Pharsight, Mountain View, CA, USA).

### RESULTS

### Retinoid extraction and HPLC retinoid separation

The analytical method used, provided the simultaneous determination of plasma concentrations of 9-*cis* RA, 4-oxo-9-*cis* RA and the internal standard 13-*cis* acitretin (Figure 1). The method showed linearity for the concentrations of 9-*cis* RA in the range of 0.01 to 25.6 μg ml$^{-1}$ plasma. The correlation coefficients for the compounds were 0.9922 and 0.9984, respectively, and the intercepts of the calibration curves did not differ significantly from zero. Each concentration was determined in duplicate according to the described method. Recovery was calculated by comparing the measured peak height of these compounds of spiked plasma to those of the standard solutions. The extraction recovery for 9-*cis* RA was 95 ± 14% and for the metabolite 83 ± 3%. All samples were analysed within 3 weeks from sampling, and repeated analysis of the same sample during this period of time yielded

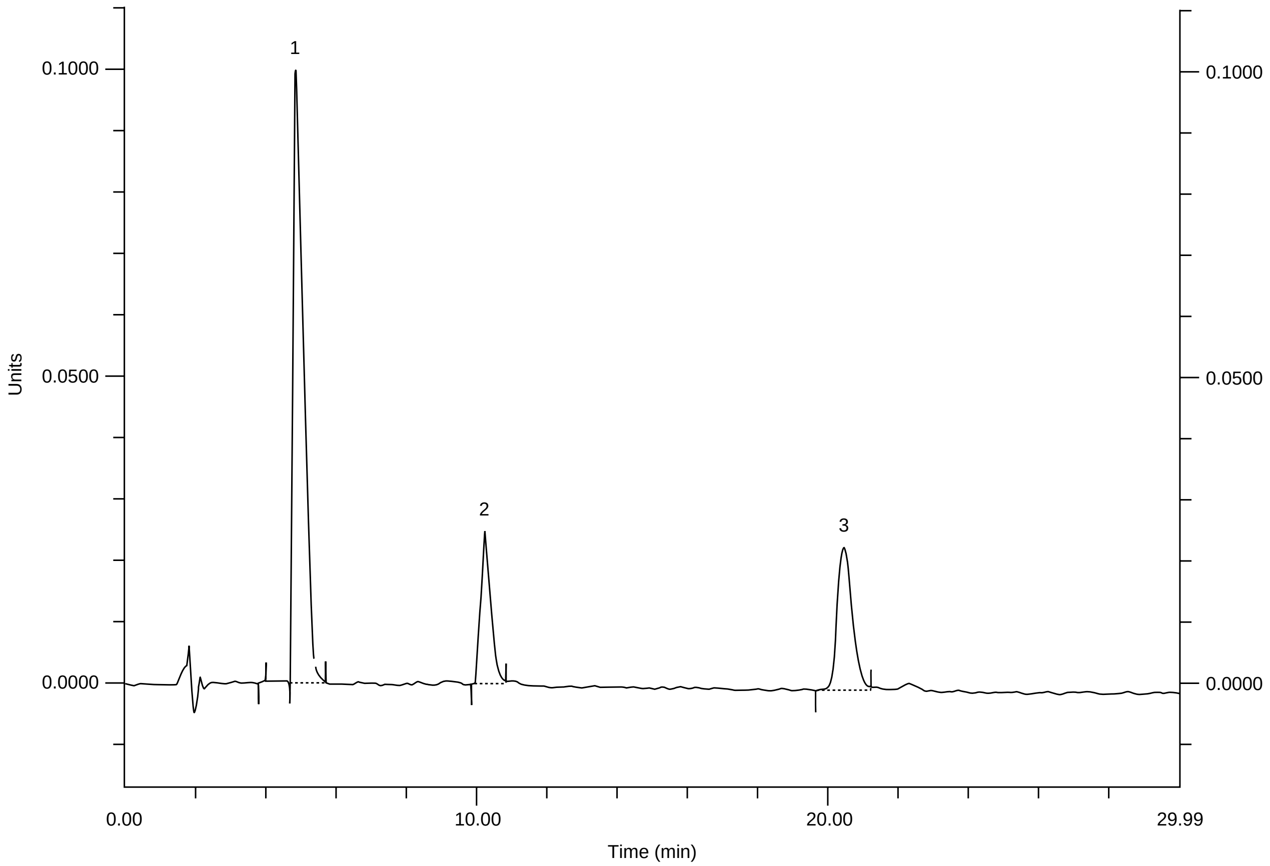

**Figure 1** Typical chromatogram obtained from a sample containing 4-oxo-9-*cis* RA, 4 μg ml$^{-1}$ (1), 13-*cis* acitretin, 1.5 μg ml$^{-1}$ (2) and 9-*cis* RA, 4 μg ml$^{-1}$ (3)

*© 2001 Cancer Research Campaign*

identical results. In additional experiments we found that the retinoid concentrations decreased considerably when stored for a longer time than 3 weeks and disappeared after more than 3 months of storage (data not shown).

### Pharmacokinetics

The pharmacokinetic parameters obtained from 9 mg 9-*cis* RA given i.v. and p.o. are compiled in Table 1. After oral administration of 9 mg 9-*cis* RA, the ratio AUC (4-oxo-9-*cis* RA)/AUC (9 *cis* RA) was 0.42 (Figure 2A). The same ratio after intravenous injection was 0.08 (Figure 2B). The bioavailability was 11% after oral administration. The elimination half-life ($t_{1/2}$) of 9-*cis* RA was 35 min (in the range 26–43 min), whereas the $t_{1/2}$ of the metabolite (4-oxo-9-*cis* RA) was 2.1 hours (in the range 2.10–2.15 h). $C_{max}$ (9-*cis* RA) after oral dose was 2996 ng $ml^{-1}$ and was reached after 1.1 hours ($T_{max}$). The metabolite had reached its maximum levels ($C_{max,i.v.}$ = 738 ng $ml^{-1}$, $C_{max,p.o.}$ = 441 ng $ml^{-1}$) after 3.9 hours (in the range 3.13–4.58 h). The distribution volume was 35.5 ml. The clearance was 0.11 ml $h^{-1}$ and 1.43 ml $h^{-1}$ for 9-*cis* RA and 4-oxo-9-*cis* RA, respectively.

### Treatment effects on tumour volume

Neuroblastoma xenografts treated with 9-*cis* RA 2 mg $day^{-1}$ showed no significant reduction in tumour volume compared to control tumours from untreated rats (Figure 3). Neuroblastoma xenografts treated with 2.5 mg of 9-*cis* RA twice daily showed a significant difference compared to corresponding controls in reduced tumour volumes at day 10 ($P < 0.05$) but not at day 8 (Figure 3).

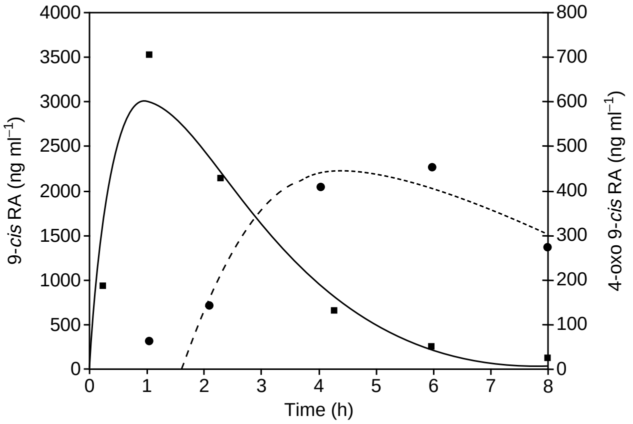

**Figure 2A** Plasma concentration-time curves of 9-*cis* RA (solid line, ■) and 4-oxo-9-*cis* RA (dashed line, ●) after one oral dose of 9 mg 9-*cis* RA

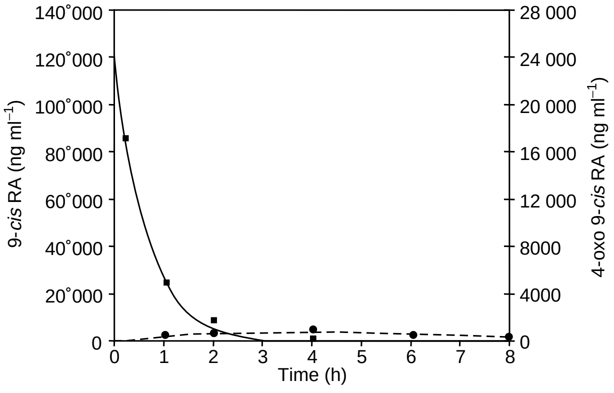

**Figure 2B** Plasma concentration-time curves of 9-*cis* RA (solid line, ■) and 4-oxo-9-*cis* RA (dashed line, ●) after one intravenous dose of 9 mg 9-*cis* RA

### Treatment effects on tumour weight

Tumours from 9-*cis* retinoic acid treated rats, regardless of treatment schedule, showed no significant reduction in tumour weight after 10–12 days of therapy compared to untreated control tumours (Figure 4). There was no significant difference between using the 2 mg $day^{-1}$ or 2.5 mg twice daily. However, there was a tendency towards a reduction in tumour weights in the 2 × 2.5 mg $day^{-1}$ treated group.

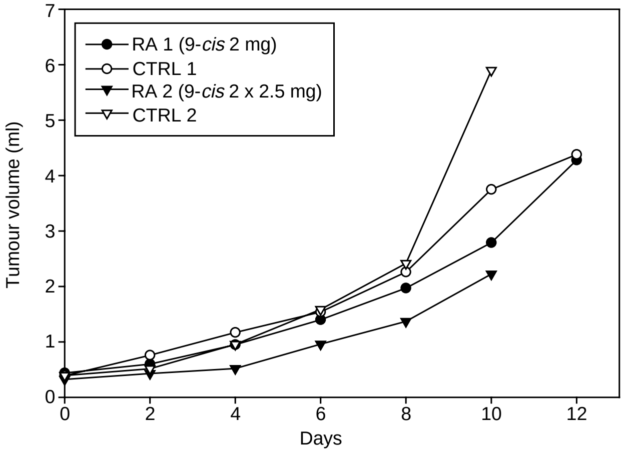

**Figure 3** Neuroblastoma SH-SY5Y xenograft tumour volume in nude rats treated with 9-*cis* RA. Mean volumes at tumour take (day 0) and 2–12 days from start of treatment in 4 different groups: tumours from, 9-*cis* RA 2 mg $day^{-1}$ treated rats (RA 1, ●), and corresponding control rats (CTRL 1, ○). Tumours from, rats treated with 9-*cis* RA 2 × 2.5 mg (RA 2, ▼), and corresponding control rats (CTRL 2, ▽). Tumour volumes for rats treated with 9-*cis* RA 2.5 mg twice daily were significantly smaller than for untreated rats at day 10 ($P < 0.05$), but not at day 8

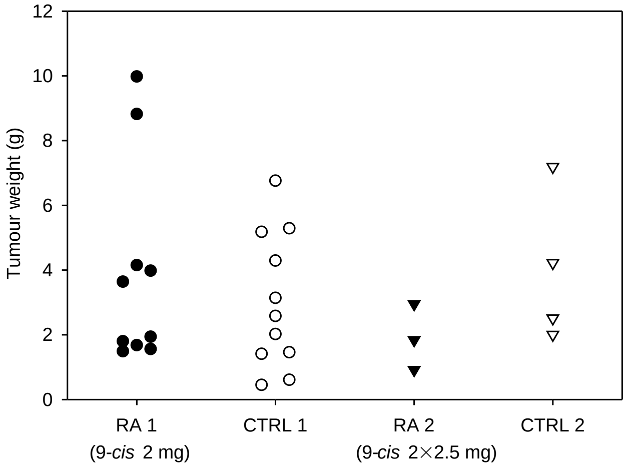

| | RA 1 | CTRL 1 | RA 2 | CTRL 2 |
|---|---|---|---|---|
| *n* | 10 | 11 | 3 | 4 |
| Median | 2.79 g | 2.58 g | 1.84 g | 3.37 g |
| Range | 1.49–9.98 g | 0.45–6.76 g | 0.92–2.95 g | 2.01–7.19 g |

**Figure 4** Neuroblastoma SH-SY5Y xenograft tumour weight at sacrifice 10–12 days from tumour take and start of 9-*cis* RA treatment in 4 different groups: tumours from 9-*cis* RA 2 mg $day^{-1}$ treated rats (RA 1, ●), and corresponding control rats (CTRL 1, ○). Tumours from 9-*cis* RA 2 × 2.5 mg treated rats (RA 2, ▼), and corresponding control rats (CTRL 2, ▽). There were no significant differences in tumour weights comparing the retinoid treated rats with untreated controls

### Toxic side effects correlated to treatment

There were no signs of any toxicity in either of the treatment groups. All animals irrespective of treatment showed a normal weight gain both before and after tumour take (day 0) (Figure 5).

### DISCUSSION

Several retinoids have shown significant effects on neuroblastoma cells in vitro and in vivo. It has also been shown that dose scheduling and toxic side effects may limit the clinical application of retinoids in children with neuroblastoma. The aim of the present study was to investigate the kinetics and the anti-tumour effect of 9-*cis* RA in order to establish the correlation between treatment efficacy and dose scheduling. There are several methods available to separate and determine retinoids (Lefebvre et al, 1995; Disdier et al, 1996; Lanvers et al, 1996). The adapted method described by us provided the sensitivity and selectivity suitable for our purposes. The use of both an internal and an external standard, made our HPLC analysing method more stable concerning alterations in the extraction procedure, surrounding temperature and fluctuations in the mobile phase. Furthermore, analysing the samples within 3 weeks prevented inaccurate results due to too long storage.

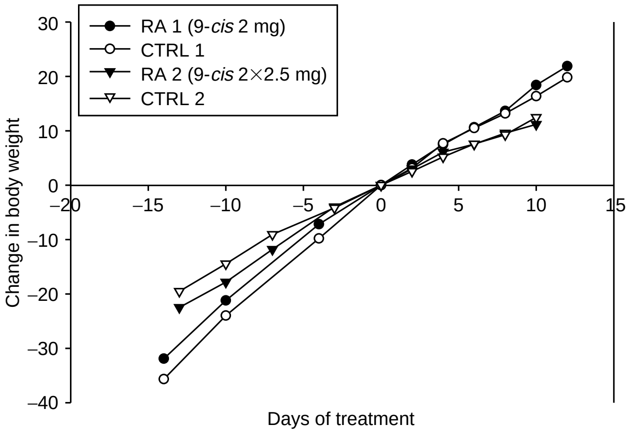

**Figure 5** The relative change in body weight during continuous treatment with 9-*cis* RA or the control treatment peanut oil. The 4 different treatment groups were: 9-*cis* RA 2 mg day$^{-1}$ treated rats (RA 1, ●), and corresponding control rats (CTRL 1, ○ ). Rats treated with 9-*cis* RA 2 × 2.5 mg (RA 2, ▼), and corresponding control rats (CTRL 2, ▽). Rats, regardless of treatment schedule, gained in weight before and during treatment

The pharmacokinetic analysis showed a low bioavailability of 9-*cis* RA (11%). We also observed that the conversion of 9-*cis* RA to its metabolite (4-oxo-9-*cis* RA) was about 8% after the intravenous dose, while the conversion was 42% after the oral dose. Hence, when comparing the AUC of the metabolite after i.v. versus p.o. administration (Table 1), the formed amount of 4-oxo-9-*cis* RA after intravenous administration was only 2-fold higher compared to that obtained after the oral dose. This may be due to a limited metabolic capacity in the liver, and/or differences in the tissue distribution after i.v. administration compared to that found after p.o. administration (Disdier et al, 2000). The low bioavailability is most probably due to the low absorption in the gastric tract, rather than first path effect. Eckhoff et al have shown that the molecular form of vitamin A and retinyl acetate did not play a major role in the metabolic transformation to more polar metabolites (Eckhoff et al, 1991). However, the authors found a higher metabolic formation rate after administration of retinoids encapsulated in detergent-based vehicles compared to oil-encapsulated vehicles. In our therapeutic study we used 9-*cis* retinoic acid solved in peanut oil, since retinoids used in the clinic are administrated in oiled based forms.

Retinoic acid, in particular 13-*cis* retinoic acid and all-*trans* retinoic acid, has been reported to induce differentiation of neuroblastoma cells under experimental conditions in vitro and in vivo (Sidell, 1982; Abemayor, 1992; Redfern et al, 1995). Other studies have shown that 9-*cis* retinoic acid can induce both differentiation and/or apoptosis in neuroblastoma cells in vitro (Redfern et al, 1995; Lovat et al, 1997). The clinical use of retinoic acid has mainly focused on treatment of minimal residual disease (MRD). 13-*cis* retinoic acid given at high-dose pulses to children with MRD of high-risk neuroblastoma, has a significant favourable therapeutic effect (Matthay et al, 1999). However, a randomised study on 13-*cis* RA given at continuous low doses to a similar group of patients demonstrated no survival advantages (Kohler et al, 2000). The preclinical and clinical data on 13-*cis* RA used against neuroblastoma indicate that dosing, scheduling, timing and tumour load at start of treatment may all be important elements in determining the therapeutic efficacy of 13-*cis* RA (Matthay and Reynolds, 2000). These elements concerning 13-*cis* RA, may also be important in developing a successful treatment of neuroblastoma with other retinoids such as 9-*cis* RA.

In our previous study we found that 3 different retinoids significantly decreased tumour growth of human SH-SY5Y neuroblastoma xenografts in nude rats (Ponthan et al, 2001). However, the high dose of 9-*cis* RA used in that study (5 mg once daily),

**Table 1** Pharmacokinetic parameters of 9-*cis* retinoic acid in rat.

| | Oral administration[a] | | Intravenous administration[a] | |
|---|---|---|---|---|
| | **9-*cis* RA** | **4-oxo-9-*cis* RA** | **9-*cis* RA** | **4-oxo-9-*cis* RA** |
| AUC[b] (ng ml$^{-1}$ h$^{-1}$) | 8548 | 3563 | 80091 | 6295 |
| $T_{1/2}$[c] (h) | 0.71 | 2.1 | 0.44 | 2.15 |
| Cl[d] (ml h$^{-1}$) | | | 0.11 | 1.43 |
| $C_{max}$[e] (ng ml$^{-1}$) | 2996 | 441 | 120476 | 738 |
| $T_{max}$[f] (h) | 1.05 | 4.58 | | 3.13 |

[a]9 mg 9-*cis* RA given p.o. or i.v. according to Materials and Methods. [b]AUC = area under the curve.
[c]$T_{1/2}$ = elimination half-life. [d]Cl = clearance. [e]$C_{max}$ = concentration maximum. [f]$T_{max}$ = time of $C_{max}$.

*© 2001 Cancer Research Campaign*

resulted in major toxic side effects, such as weight loss, yellowish colour, dry skin and diarrhoea. The main focus of our present study was to analyse the bioavailability of 9-*cis* RA. Furthermore, we investigated whether changes in doses and treatment scheduling could reduce the toxic side effects with retained tumour-inhibiting effect. For these in vivo studies we used individual control groups for each therapeutic experiment to eliminate the possible differences in tumour growth which may be caused by environmental alterations concerning cell culturing and animal breeding.

When the daily dose of 9-*cis* RA was reduced to 2 mg $day^{-1}$, no signs of toxicity were observed (Figure 5). However, the tumour growth-inhibiting effect was not significant compared to corresponding placebo-treated controls. By altering the scheduling of the toxic but tumour effective dose 5 mg $day^{-1}$ by giving 2.5 mg twice daily no signs of toxicity could be detected (Figure 5). The absence of toxicity after dividing the daily dose indicates that the toxicity from 9-*cis* RA is mainly depending on the peak concentration ($C_{max}$). The altered scheduling resulted in a limited but statistically significant reduction in tumour growth, in terms of tumour volume at end of treatment (Figure 3). Tumour weights showed no significant difference when comparing the group treated with 9-*cis* RA 2.5 mg twice daily to the corresponding untreated control tumours (Figure 4). Because of the tendency towards a reduction in tumour weights we compared the $2 \times 2.5$ mg $day^{-1}$ treated tumours with all control tumours taken together. However, this statistical comparison did not show a significant difference in tumour weights. The limited effects, if any, on both tumour volumes and tumour weights using the divided dose could argue that the AUC of 9-*cis* RA only has a limited influence on the tumour inhibiting effect of the retinoid. It seems likely that the peak concentration ($C_{max}$) is important for the tumour growth inhibiting effect of 9-*cis* RA.

Combining the results from these experiments on 3 different treatment schedules used in the current and the previous study (Ponthan et al, 2001), we conclude that 9-*cis* RA may reduce tumour growth in vivo in a dose-dependent manner. However, the toxicity profile, the short half-life and the low bioavailability of 9 *cis* RA in vivo, limit the potential use for clinical oral therapy of neuroblastoma in children.

### ACKNOWLEDGEMENT

This work was supported in part by the Swedish Children's Cancer Foundation, Cancer Society of Stockholm and Märta and Gunnar V Philipson Foundation.

### REFERENCES

Abemayor E (1992) The effects of retinoic acid on the in vitro and in vivo growth of neuroblastoma cells. *Laryngoscope* **102**: 1133–1149

Anzano MA, Byers SW, Smith JM, Peer CW, Mullen LT, Brown CC, Roberts AB and Sporn MB (1994) Prevention of breast cancer in the rat with 9-cis-retinoic acid as a single agent and in combination with tamoxifen. *Cancer Res* **54**: 4614–4617

Biedler JL, Helson L and Spengler BA (1973) Morphology and growth, tumorigenicity, and cytogenetics of human neuroblastoma cells in continuous culture. *Cancer Res* **33**: 2643–2652

Disdier B, Bun H, Catalin J and Durand A (1996) Simultaneous determination of all-trans-, 13-cis-, 9-cis-retinoic acid and their 4-oxo-metabolites in plasma by high-performance liquid chromatography. *J Chromatogr B Biomed Appl* **683**: 143–154

Disdier B, Marchetti MN, Bun H, Placidi M and Durand A (2000) Kinetics of plasma and tissue distribution of 9-cis-retinoic acid in rat. *Skin Pharmacol Appl Skin Physiol* **13**: 9–16

Eckhoff C, Bailey JR, Collins MD, Slikker W Jr. and Nau H (1991) Influence of dose and pharmaceutical formulation of vitamin A on plasma levels of retinyl esters and retinol and metabolic generation of retinoic acid compounds and beta-glucuronides in the cynomolgus monkey. *Toxicol Appl Pharmacol* **111**: 116–127

Heyman RA, Mangelsdorf DJ, Dyck JA, Stein RB, Eichele G, Evans RM and Thaller C (1992) 9-cis retinoic acid is a high affinity ligand for the retinoid X receptor. *Cell* **68**: 397–406

Howell SR, Shirley MA and Ulm EH (1998) Effects of retinoid treatment of rats on hepatic microsomal metabolism and cytochromes P450. Correlation between retinoic acid receptor/retinoid × receptor selectivity and effects on metabolic enzymes. *Drug Metab Dispos* **26**: 234–239

Kohler JA, Imeson J, Ellershaw C and Lie SO (2000) A randomized trial of 13-Cis retinoic acid in children with advanced neuroblastoma after high-dose therapy. *Br J Cancer* **83**: 1124–1127

Lanvers C, Hempel G, Blaschke G and Boos J (1996) Simultaneous determination of all-trans-, 13-cis- and 9-cis-retinoic acid, their 4-oxo metabolites and all-trans-retinol in human plasma by high-performance liquid chromatography. *J Chromatogr B Biomed Appl* **685**: 233–240

Lefebvre P, Agadir A, Cornic M, Gourmel B, Hue B, Dreux C, Degos L and Chomienne C (1995) Simultaneous determination of all-trans and 13-cis retinoic acids and their 4-oxo metabolites by adsorption liquid chromatography after solid-phase extraction. *J Chromatogr B Biomed Appl* **666**: 55–61

Lovat PE, Irving H, Annicchiarico-Petruzzelli M, Bernassola F, Malcolm AJ, Pearson AD, Melino G and Redfern CP (1997) Apoptosis of N-type neuroblastoma cells after differentiation with 9-cis-retinoic acid and subsequent washout. *J Natl Cancer Inst* **89**: 446–452

Matthay KK and Reynolds CP (2000) Is there a role for retinoids to treat minimal residual disease in neuroblastoma? *Br J Cancer* **83**: 1121–1123

Matthay KK, Villablanca JG, Seeger RC, Stram DO, Harris RE, Ramsay NK, Swift P, Shimada H, Black CT, Brodeur GM, Gerbing RB and Reynolds CP (1999) Treatment of high-risk neuroblastoma with intensive chemotherapy, radiotherapy, autologous bone marrow transplantation, and 13-cis-retinoic acid. Children's Cancer Group. *N Engl J Med* **341**: 1165–1173

Nilsson S, Pahlman S, Arnberg H, Letocha H and Westlin JE (1993) Characterization and uptake of radiolabelled meta-iodobenzylguanidine (MIBG) in a human neuroblastoma heterotransplant model in athymic rats. *Acta Oncol* **32**: 887–891

Ponthan F, Borgstrom P, Hassan M, Wassberg E, Redfern CP and Kogner P (2001) The vitamin A analogues: 13-cis retinoic acid, 9-cis retinoic acid, and Ro 13-6307 inhibit neuroblastoma tumour growth in vivo. *Med Pediatr Oncol* **36**: 127–131

Redfern CP, Lovat PE, Malcolm AJ and Pearson AD (1995) Gene expression and neuroblastoma cell differentiation in response to retinoic acid: differential effects of 9-cis and all-trans retinoic acid. *Eur J Cancer* **31**: 486–494

Reynolds CP, Kane DJ, Einhorn PA, Matthay KK, Crouse VL, Wilbur JR, Shurin SB and Seeger RC (1991) Response of neuroblastoma to retinoic acid in vitro and in vivo. *Prog Clin Biol Res* **366**: 203–211

Shirley MA, Bennani YL, Boehm MF, Breau AP, Pathirana C and Ulm EH (1996) Oxidative and reductive metabolism of 9-cis-retinoic acid in the rat. Identification of 13,14-dihydro-9-cis-retinoic acid and its taurine conjugate. *Drug Metab Dispos* **24**: 293–302

Sidell N (1982) Retinoic acid-induced growth inhibition and morphologic differentiation of human neuroblastoma cells in vitro. *J Natl Cancer Inst* **68**: 589–596

Sucov HM and Evans RM (1995) Retinoic acid and retinoic acid receptors in development. *Mol Neurobiol* **10**: 169–184

Wassberg E, Pahlman S, Westlin JE and Christofferson R (1997) The angiogenesis inhibitor TNP-470 reduces the growth rate of human neuroblastoma in nude rats. *Pediatr Res* **41**: 327–333

United Kingdom Co-ordinating Committee on Cancer Research (UKCCCR) (1998) Guidelines for the Welfare of Animals in Experimental Neoplasia (Second Edition). *Br J Cancer* **77**: 1–10

*© 2001 Cancer Research Campaign*
